# Transfemoral TAVR in a Quadricuspid Aortic Valve With Pure Regurgitation: Overcoming Anatomical and Technical Barriers

**DOI:** 10.1016/j.jaccas.2025.104879

**Published:** 2025-09-03

**Authors:** Julien Feghaly, Valentin Suma, Fadi Kandah, Calvin Choi, Ali Zgheib

**Affiliations:** Division of Cardiology, Department of Medicine, University of Florida Health, Jacksonville, Florida, USA

**Keywords:** aortic regurgitation, quadricuspid aortic valve, transcatheter aortic valve replacement

## Abstract

We present a case of successful transfemoral transcatheter aortic valve replacement for a rare quadricuspid aortic valve with pure aortic regurgitation. Procedural challenges included identifying the annular plane, determining the appropriate deployment view, annular sizing, the absence of annular calcium, and the lack of a dedicated transcatheter device for pure aortic regurgitation.

## Graphical Abstract

**Figure:**
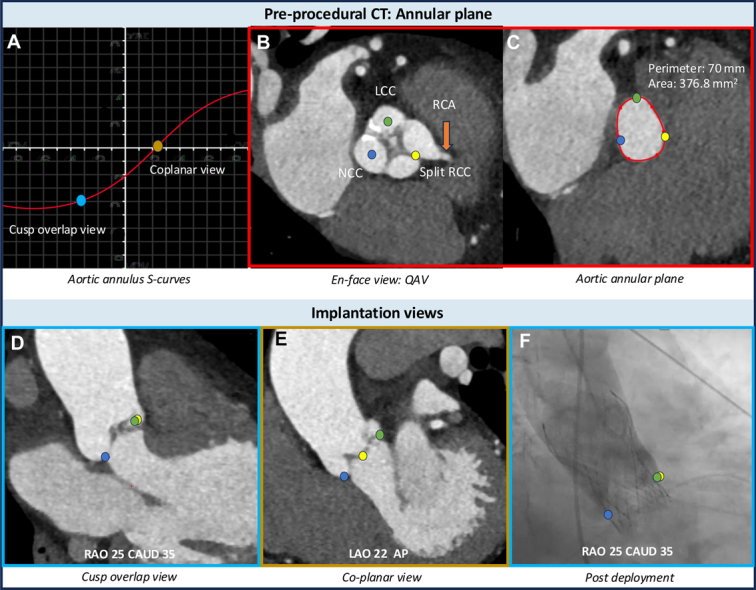

## Background

Quadricuspid aortic valve (QAV) is a rare cardiac variant with an incidence of 0.008% to 0.043%.^1^ QAV with isolated aortic regurgitation poses significant challenges for off-label transcatheter aortic valve replacement due to valve sizing and deployment,^2^ owing to a lack of annular calcium and risk of embolization or paravalvular leak.^3^

**Take-Home Messages**

• Transcatheter aortic valve replacement in QAV with pure regurgitation remains technically challenging due to lack of calcium, complex cusp anatomy, and absence of a dedicated device.
• This case highlights how individualized computed tomography planning, cusp-overlap view selection, and use of a recapturable oversized valve can overcome these barriers and lead to successful outcomes.

## Clinical Vignette

A 69-year-old woman with diabetes mellitus, hypertension, normal coronary arteries, and chronic kidney disease presented with progressive exertional dyspnea. Transthoracic and transesophageal echocardiography revealed a rare QAV with severe aortic regurgitation (Videos 1 and 2) and left ventricular enlargement with preserved systolic function. The patient was deemed a poor surgical candidate due to significant comorbidities and frailty.

Cardiac computed tomography demonstrated an asymmetric QAV with minimal leaflet calcification, an annular perimeter of 70 mm, and sinus of Valsalva diameters ranging from 30 to 31 mm. After heart team discussion, a transfemoral TAVR was performed using a self-expanding 29 mm Medtronic Evolut FX+ valve (Video 3).

**Figure 1:**
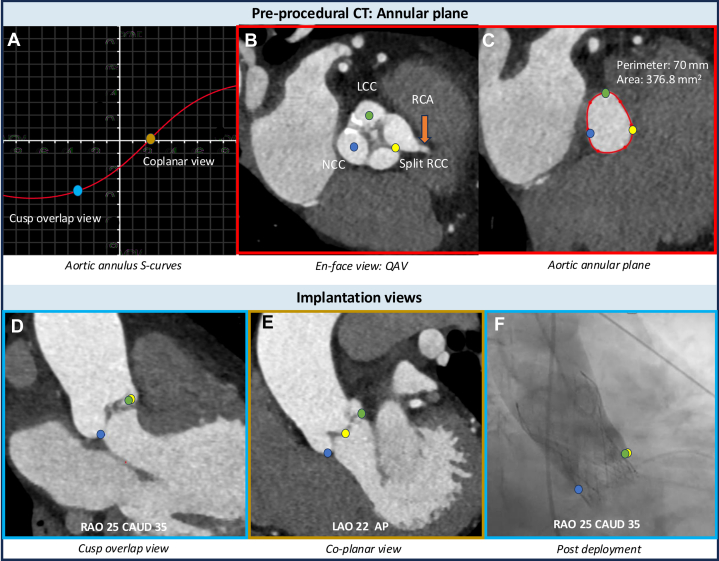
CT-Guided Procedural Planning and Fluoroscopic Angles in Quadricuspid Aortic Valve (A) CT-derived aortic annulus S-curve (red), with cusp-overlap view (blue dot) and co-planar view (brown dot). (B) En face view of the aortic valve showing left coronary cusp (LCC, green), non-coronary cusp (NCC, blue), and right coronary cusp (RCC, yellow); the RCC was functionally treated as a single cusp despite being split, to facilitate obtaining the annular plane. (C) Nadir points of the LCC, NCC, and split RCC used to obtain the annular plane. (D) Cusp-overlap view (RAO 25°, caudal 35°) isolating the NCC and overlapping LCC and RCC for deployment depth guidance. (E) Co-planar view (LAO 22°, AP) aligning all 3 cusps in a single plane. (F) Final valve deployment performed in cusp-overlap view (RAO 25°, caudal 35°) using the NCC as depth reference. CAUD = caudal; LAO = Left anterior oblique; LCC = left coronary cups; NCC = non-coronary cusp; RAO = right anterior oblique; RCC = right coronary cups.

In QAV anatomy, the absence of calcification and 4 asymmetric cusps make obtaining the annular plane through cusps nadir alignment challenging, complicating fluoroscopic guidance for valve deployment. In our case, preprocedural computed tomography analysis was optimized by functionally treating the split right coronary cusp as a single cusp to facilitate finding the annular plane and implantation view. Given the limitations of coplanar views in the setting of 4 cusps, a double cusp-overlap projection was used to isolate the noncoronary cusp and overlap the remaining cusps, providing a reliable fluoroscopic reference for valve positioning (Figure 1). The lack of significant annular calcium increased the risk of embolization and paravalvular leak; therefore, a 29 mm self-expanding Evolut FX+ valve was selected for its recapturability and oversizing (30% in this case). Echocardiography confirmed a well-seated valve without residual regurgitation or leak (Videos 4 and 5).

## Funding Support and Author Disclosures

The authors have reported that they have no relationships relevant to the contents of this paper to disclose.

## Appendix

Video 1 Transesophageal Echocardiography (TEE), Long-Axis (LVOT) View Demonstrating Torrential Aortic Regurgitation

Video 2 TEE Short-Axis View Revealing a QAV With 4 Separate Cusps

Video 3 Fluoroscopic Imaging During Deployment of a 29 mm Evolut FX + Valve in the Cusp-Overlap View, Showing Appropriate Depth and Alignment

Video 4 Transthoracic Echocardiography (TTE), Parasternal Long-Axis View Confirming Optimal Valve Positioning With no Residual Regurgitation

Video 5 TTE, Parasternal Short-Axis View Showing a Well-Expanded Transcatheter Valve in Situ
